# Psychiaterinnen im Film: Ursprung und Entwicklung eines genderspezifischen Figurenstereotyps (1935–2020)

**DOI:** 10.1007/s00115-025-01901-y

**Published:** 2025-09-25

**Authors:** Dennis Henkel, Walter Stehling, Katja Wagemans

**Affiliations:** 1 Institut für Geschichte und Ethik der Medizin, Universität zu Köln, Medizinische Fakultät und Universitätsklinikum Köln, Joseph-Stelzmann-Str. 20, 50931 Köln, Deutschland; 2 Tagesklinik Süd für Psychiatrie und Psychotherapie, Bayerisches Rotes Kreuz, Kreisverband München, München, Deutschland; 3 Klinik für Kinder- und Jugendpsychiatrie, -psychotherapie und -psychosomatik im UKE, Hamburg, Deutschland

**Keywords:** Psychiatrie im Film, Psychiatriegeschichte, Filmgeschichte, Geschlechterunterschiede, Geschichte der Medizin, Psychiatry in films, Psychiatry/history, Motion pictures/history, Gender differences, History of medicine

## Abstract

**Hintergrund:**

In der Medizinwelt haben Frauen über die letzten Jahrzehnte eine stetig größere Rolle eingenommen und sind in vielen Fachgebieten den Männern mittlerweile numerisch überlegen. Die Psychiatrie bildet hier keine Ausnahme. Da die Filmkunst stets gesellschaftliche Entwicklungen spiegelt, stellt sich die Frage, ob sich dieser Trend auch in Film und Serie abzeichnet.

**Methodik:**

Inszenierungen von Psychiaterinnen-Figuren wurden systematisch anhand einer gezielten Suche in relevanten Datenbanken und mithilfe ergänzender Literaturrecherchen nachgewiesen. Zusätzlich erfolgte eine Analyse einschlägiger Filmbeispiele bis ins 21. Jahrhundert.

**Fragestellung:**

Im Mittelpunkt stehen sozial-, medizin- und filmhistorisch relevante Forschungsfragen: Entspricht das Bild der Leinwand-Psychiaterinnen der Realität? Werden Stereotypen bedient bzw. initiiert? Wie unterscheidet sich die Darstellung vom männlichen Pendant?

**Ergebnisse und Diskussion:**

Die exemplarische Untersuchung von 18 Filmen sowie einer Serie offenbart die frühe Entstehung eines bis heute einflussreichen, genderspezifischen Figurenklischees: die Psychiaterin als empathisch und sensibel agierende Ärztin, die kraft dieser Charaktereigenschaften zwar Karriere macht, dadurch aber emotional und sozial ins Abseits gerät. Eine chronologisch gegliederte Analyse legt nicht nur gesellschaftliche Prägungen dieses Stereotyps offen, sondern zeigt auch Mechanismen der medialen Tradierung auf. Eine Reflexion dieser Prozesse kann in professionellen Settings helfen, medial induzierte Vorbehalte bei Patientinnen und Patienten zu erkennen und gezielt zu bearbeiten.

## Abstract (en)

**Background:**

In the medical world women have taken on a steadily increasing role over the last decades and are now numerically superior to men in many specialized fields. In this context psychiatry is no exception. As cinematics always reflect social developments, this fact raises the question of whether the representation of female psychiatrists in films and series takes this trend into account.

**Methods:**

Film productions presenting female psychiatrist characters were systematically identified by means of a targeted search in relevant databases and a supplementary literature search. In addition, pertinent film examples up to the twenty-first century were analyzed.

**Objective:**

The focus is on research questions relevant to social, medical and film history: Does the image of screen psychiatrists correspond to reality? Are stereotypes served or initiated? How does the portrayal differ from the male counterpart?

**Results and conclusion:**

The exemplary examination of 18 films and 1 series reveals the early emergence of a gender-specific character cliché which is still influential today: the female psychiatrist as an empathic, sensitive and emotionally active doctor who can make a successful career by virtue of these character traits but who is also emotionally and socially marginalized as a result. A chronologically structured analysis not only reveals social contexts of this stereotype but also shows mechanisms of media transmission. Reflecting on this distorted image in professional settings can help to recognize and specifically address media-induced reservations in patients.

Das Massenmedium Film erreicht seit Beginn des 20. Jahrhunderts alle sozialen Schichten und übt nachhaltigen Einfluss auf die Meinungsbildung des Publikums aus. Dies gilt auch für das Image der Medizinwelt: Ist die Zuschauerschaft groß genug, erstreckt sich die Wirkung der Filmkunst auf weite Teile der Gesellschaft und kann den Ruf und das Ansehen von Krankheiten, Patienten, Medizinern und Therapien nachhaltig verändern. Vor diesem Hintergrund scheint die Überlegung bedeutsam, welches „Bild“ die Kinematographie von der weiblichen Ärzteschaft entwirft.

Die Psychiatrie stellt den mit Abstand häufigsten medizinischen Topos der Filmgeschichte [12] dar und die Figur des Psychiaters hat sich als Gegenstand interdisziplinärer Forschung etabliert. Schon 1945 wurde eine Stereotypen-Trias entworfen, die seither Schule gemacht hat: der abgrundtief böse „Dr. Evil“, sein der Verklärung anheimfallendes Gegenstück „Dr. Wonderful“ und der liebenswert-lächerliche „Dr. Dippy“ [10, 14, 25]. Diese ausschließlich männliche Rollentypisierung wirft spannende Forschungsfragen auf: Werden auch Charaktere von Psychiaterinnen typisiert? Sind verbindende Gemeinsamkeiten der Figurenkonstruktionen nachweisbar, oder ist das weibliche Pendant (bzw. dessen jahrzehntelange Vernachlässigung) gar ein Produkt patriarchalischer Gesinnung der Filmindustrie? Bedient oder initiiert die Inszenierung von Therapeutinnen gar genderspezifische Vorurteile?

An dieser Stelle sei explizit angemerkt, dass die Verwendung der Begriffe „Stereotyp“, „Klischee“, „Typus“ oder „Zerrbild“ nicht die Existenz einer „richtigen Rolle“ für Psychiaterinnen implizieren soll. Der Wortgebrauch übernimmt vielmehr – aus Gründen wissenschaftlicher Kohärenz und Vergleichbarkeit – die Verwendung jener Terminologie aus o. g. Forschungsarbeiten.

## Methodik

Um diese Fragen zu beantworten, wurden Filme, in denen Psychiaterinnen tragende Rollen bekleiden oder als Nebendarstellerinnen handlungsrelevante Funktionen einnehmen, mittels umfassender Literatur- und Datenbankrecherche [1, 2, 4, 5, 7, 9, 17, 21, 22, 27] systematisch identifiziert. Als wesentliche Schlagworte wurden u. a. „psychiatrist“, „therapist“, „psychologist“, „physician“, „psychotherapy“ oder „psychoanalysis“ in Kombination mit „female“ in Deutsch und Englisch verwendet. Psychologische Psychotherapeutinnen wurden eingeschlossen, da die Abgrenzung zu ärztlichen Berufsbildern in den Filmen oft unscharf ist. Methodisch wurden zwei verschiedene Vorgehensweisen kombiniert:

1. Bei vor 1960 entstandenen Produktionen wurde jedes zu eruierende Werk mit Psychiaterinnen-Figuren berücksichtigt, um eine umfassende Chronologie der Entstehung der Ärztinnen-Figuren nachzuzeichnen.
2. Aufgrund stark ansteigender Produktionszahlen war eine chronologisch lückenlose Aufarbeitung aller Psychiaterinnen-Figuren für die Jahrzehnte ab 1960 nicht mehr möglich. Stattdessen wurden entsprechende Darstellungen in exemplarisch ausgewählten, publikumswirksamen Produktionen analysiert und mit den Befunden der frühen Epoche verglichen. Publikumswirksam bedeutet hier, dass die Werke ein möglichst großes Publikum fanden – sog. „Blockbuster“ – und über die Kinokassen hinaus durch internationale Streaming- und Home-Video-Vermarktung breite Schichten der Bevölkerung erreichten.
  Diese Selektion ermöglicht die Annahme einer relevanten Beeinflussung der gesellschaftlichen Meinungsbildung, bedingt jedoch zugleich eine Vernachlässigung kleinerer und internationaler Produktionen, die Gegenstand zukünftiger Forschungsarbeiten sein sollten. So entstand das epochenübergreifende Bild einer Rolle, die sich in annähernd 100 Jahren Filmgeschichte entwickelte (Tab. 1).

**Tab. 1:** Chronologie der für diesen Aufsatz ausgewählten Werke mit Angabe der Darstellerinnen

Originaltitel	Produktionsland	Jahr	Regie	Darstellerin der Psychiaterin
Cupid vs. Cigarettes	USA	1912	Unbekannt	Unbekannt
Private Worlds	USA	1935	Gregory La Cava	Claudette Colbert
Spellbound	USA	1945	Alfred Hitchcock	Ingrid Bergman
High Wall	USA	1947	Curtis Bernhardt	Audrey Totter
Let’s Live a Little	USA	1948	Richard Wallace	Hedy Lamarr
Shadow on the Wall	USA	1950	Gregory La Cava	Nancy Reagan
Knock on Wood	USA	1954	Melvin Frank u. Norman Panama	Mai Zetterling
Susan Slept Here	USA	1954	Frank Tashlin	Rita Johnson
Sex and the Single Girl	USA	1964	Richard Quine	Natalie Wood
Zelig	USA	1983	Woody Allen	Mia Farrow
Agnes of God	USA	1985	Norman Jewison	Jane Fonda
Lethal Weapon	USA	1987	Richard Donner	Mary Ellen Trainor
Lethal Weapon 2	USA	1989	Richard Donner	Mary Ellen Trainor
Herr der Gezeiten	USA	1991	Barbra Streisand	Barbra Streisand
Basic Instinct	USA, FR	1991	Paul Verhoeven	Jeanne Tripplehorn
Lethal Weapon 3	USA	1992	Richard Donner	Mary Ellen Trainor
Mr. Jones	USA	1992	Mike Figgis	Lena Olin
Batman Forever	USA	1995	Joel Schumacher	Nicole Kidman
Lethal Weapon 4	USA	1998	Richard Donner	Mary Ellen Trainor
The Sopranos	USA	1999–07	Diverse	Lorraine Bracco

Allein bis zum Jahr 2000 lassen sich 38 solcher Werke nachweisen [18], in den letzten 2 Dekaden kamen weitere 53 Produktionen hinzu [19]. Betrachtet man die Anzahl zugelassener Psychiaterinnen in den USA, zeigt sich ein vergleichbarer Trend: Waren von 1950 bis 1970 nur etwa 5 % des Berufsstandes weiblich, stieg die Anzahl in den 1980er-Jahren auf 20 %, und im neuen Jahrtausend auf 42 % [16].

## Ergebnisse

### Die Anfänge (1912–1960)

Während der männliche Seelenheiler schon im Stummfilm über die Leinwand flackerte – 1906 erschien das komödiantische Werk *Dr. Dippy’s Sanitarium* (USA, Regie unbekannt), das namengebend für das dritte der o. g. Stereotype wurde –, lässt sich für das stumme Kino nicht eine einzige Psychiaterinnen-Figur nachweisen.

**Abb. 1:**
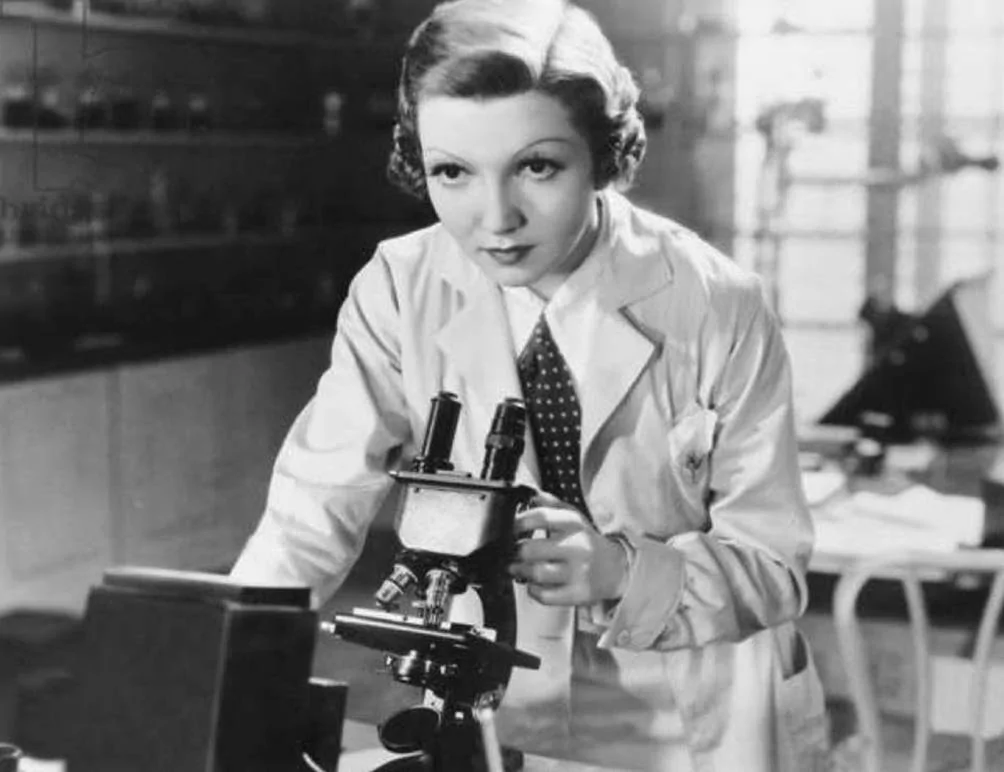
Die erste Psychiaterin der Filmgeschichte wird mit einem Mikroskop abgelichtet, um die wissenschaftliche Kompetenz der Ärztin beim Publikum deutlich zu machen. (Aus *Private Worlds*.)

Die erste Rolle einer Psychiaterin erscheint erst 1935 im Drama *Private Worlds* (USA 1935, Gregory La Cava) mit hochkarätiger Besetzung: Claudette Colbert (1903–1996) spielt eine kompetente und progressive Ärztin (Abb. 1), die auf einen zutiefst konservativen Mann trifft, der beruflich engagierte und erfolgreiche Frauen dezidiert ablehnt. Schnell wird aus dem scheinbar konträren Duo jedoch ein Paar, die Ärztin stellt ihre Karriere zugunsten der entflammenden Romanze zurück und ein sich anbahnendes Familienleben bildet das Happy End. Bemerkenswert erscheint dabei die Figurenzeichnung der Medizinerin: Einfühlungsvermögen (als typisch weiblicher Wesenszug inszeniert) wird als Haupteigenschaft der Protagonistin präsentiert und begründet sowohl ihren beruflichen Erfolg als auch ihre „unprofessionelle“ Verliebtheit. Das Werk skizziert ein Singledasein als Schicksalsschlag und Karrierestreben als unnötig anstrengende „Anomalie“. Die Aussicht auf Ehestand und Familienglück bringen das Leben der Medizinerin am Ende jedoch wieder ins Lot.

**Abb. 2:**
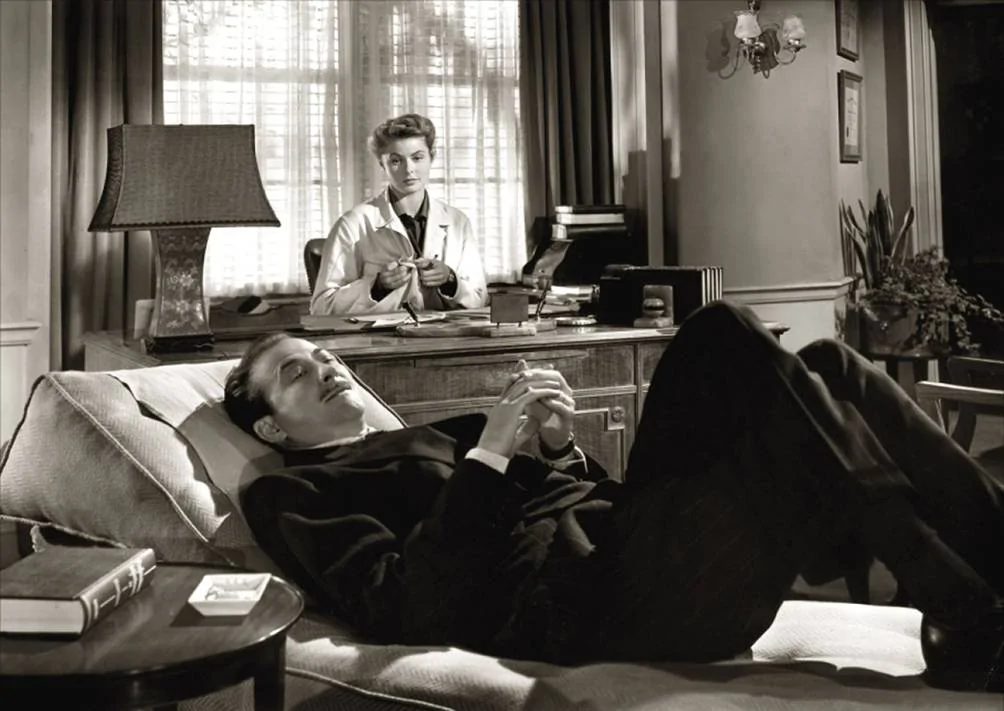
Ein Beispiel für die klassische Couchszene, in der die Therapeutin (hier gespielt von Ingrid Bergmann) hinter dem liegenden Patienten positioniert ist. (Aus *Spellbound*.)

Dieses Muster wiederholt sich 10 Jahre später im Klassiker *Spellbound* (USA 1945, Alfred Hitchcock). Dr. Constance Petersen (Ingrid Bergman) verliebt sich in einen Patienten (siehe Abb. 2), der unter Mordverdacht steht. Erneut erscheint die vermeintlich feminine Empathiefähigkeit als Grund sowohl für das berufliche Ansehen der Ärztin als auch für ihre Risikobereitschaft in puncto Beziehungen. Ein Leben als Karrierefrau wirkt wie eine glücklose Existenz, die nur durch eine amouröse Verbindung zum anderen Geschlecht zu „retten“ ist.

Der Charakterentwurf der ersten beiden Film-Psychiaterinnen hält sich bis ins Jahr 1960: In *High Wall *(USA 1947, Curtis Bernhardt) glaubt eine erfolgreiche Gefängnispsychiaterin an die Unschuld eines geständigen Mörders, verliebt sich der Gefahr zum Trotz in ihn und wird schließlich seine Partnerin. *Let’s Live a Little *(USA 1948, Richard Wallace) zeigt einen bevormundend auftretenden Patienten mit der Überzeugung, Frauen seien „unfähig Geschäftliches von Emotionalem zu trennen“. Doch gewinnt er die Liebe der Ärztin und bekehrt die Karrierefrau. In *Knock on Wood* (USA 1954, Melvin Frank und Norman Panama) therapiert der Kranke sogar seinerseits die Medizinerin. Und auch hier werden die beiden ein Paar.

Eine gewisse Ausnahme bildet *Susan Slept Here* (USA 1954, Frank Tashlin): Zwar gibt die Ärztin dem Protagonisten einen Ratschlag, der die Handlung maßgeblich vorantreibt, jedoch bekleidet die insgesamt blasse Psychiaterinnen-Figur hier bloß eine Nebenrolle mit wenigen Minuten Leinwandzeit. Allein *Shadow on the Wall* (USA 1950, Gregory La Cava) präsentiert eine Therapeutin in der Hauptrolle außerhalb des Mainstreammusters: Die von Nancy Davis (später Nancy Reagan [1921–2016]) dargebotene Medizinerin entlastet einen zu Unrecht verurteilten Mörder, ohne dass die Liebe, das Geschlecht oder die Eignung von Frauen für den Arztberuf überhaupt zum Thema werden – ein in dieser Hinsicht ausgesprochen fortschrittliches Melodram.

Festzuhalten bleibt: Die meisten der vor 1960 entstandenen Werke mit einer Psychiaterin als Protagonistin entwerfen ein Figurenstereotyp, das sich stark vom männlichen Gegenstück unterscheidet und durch folgende Merkmale auszeichnet:

1. Die Ärztinnen wirken kompetent und erfolgreich, müssen sich aber stets behaupten. Primär weiblich verstandene Eigenschaften (Empathie, Emotionalität, Kommunikationsfähigkeit) bedingen den Karriereerfolg.
2. Die Berufstätigkeit einer Singlefrau wird als Sonderfall dargestellt, der ein erfülltes Leben samt Familienglück verhindert. Frauen können demnach durchaus als kompetente Psychiaterinnen agieren und die Karriereleiter erklimmen, jedoch nur entgegen ihrer „weiblichen Natur“, was ins Unglück führt.
3. Der männliche Charakter, stets konservativ und aufstrebenden Frauen gegenüber ablehnend eingestellt, erobert das Herz der Psychiaterin. Dieselben Eigenarten, die ihren professionellen Aufstieg bedingen, führen – sobald die Liebe ins Spiel kommt – zu unmoralischem und unprofessionellem Handeln. Karrierefrau zu sein, so der Tenor des Filmkorpus, ist ein abnormer Zustand, aus dem nur ein Mann die Fehlgeleitete erlösen kann. Sie entwickelt sich zu „Dr. Correction“.

Auch motivischer Fokus und Genrespektrum fallen auf: Die Romanze steht im Mittelpunkt, sei es im Kontext von Komödien oder Thrillern. Die Plots demonstrieren, welch belustigende Wirkung die Vorstellung einer erfolgreichen Psychiaterin bei Filmemachern und Publikum haben musste.

### Vom Klischee zum Mainstream (1960–2000)

In den darauffolgenden 4 Jahrzehnten wandelte sich das Image der Seelenärztinnen zwar nicht entscheidend, doch es kam immerhin zu einer allmählichen Differenzierung und vor allem Popularisierung. Das 1964 erschienene Drama *Sex and the Single Girl* (USA, Richard Quine) war mit Tony Curtis (1925–2010) und Natalie Wood (1938–1981) zwar ansprechend besetzt, doch bediente es sich hinsichtlich Klischees und Figurenkonstellationen bei früheren Werken:

Die empathische Psychiaterin begegnet dem charmanten Patienten, dem es im Handlungsverlauf gelingt, ihr Herz zu erobern. Der feministisch ambitionierte Handlungsrahmen verliert jedoch zwischen Klamauk und Aberwitz seine Relevanz und der glückliche Schluss wird allein durch die Liaison von Ärztin und Patient bestimmt.

**Abb. 3:**
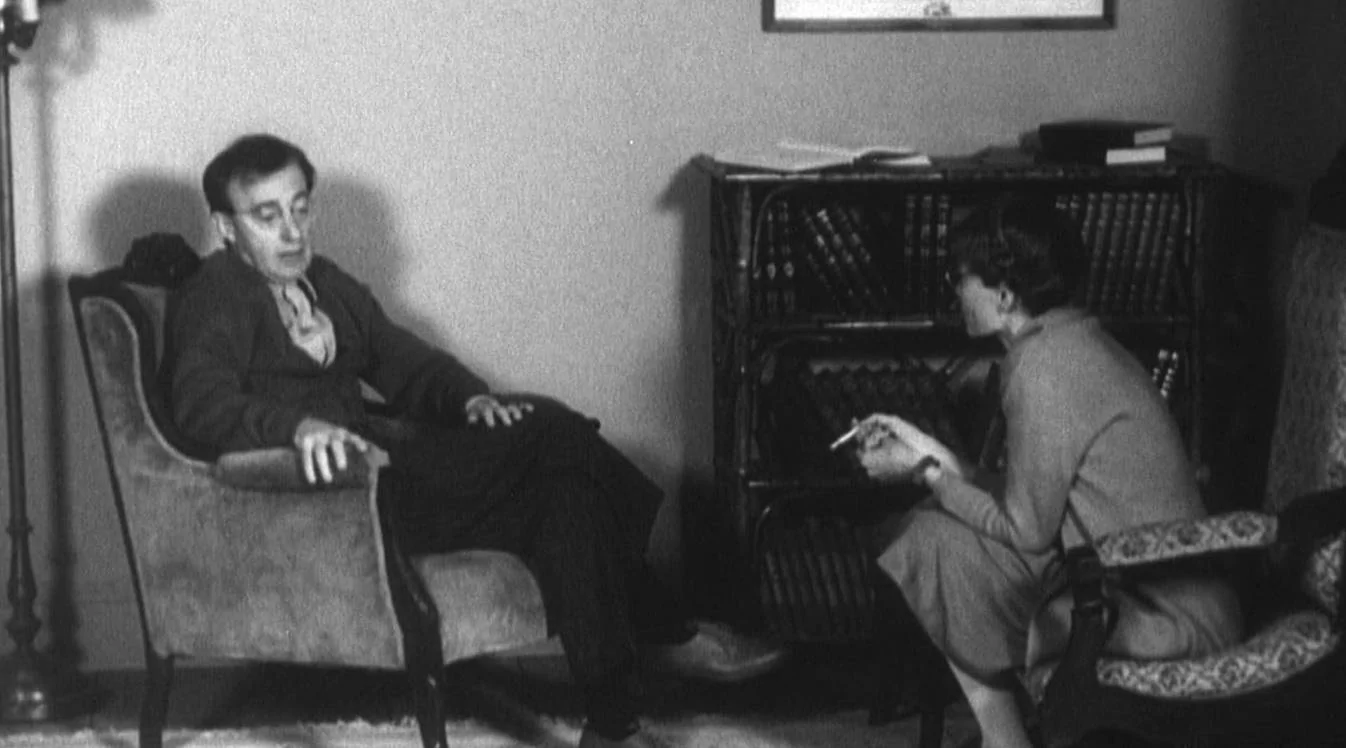
Die Therapeutin (gespielt von Diane Keaton) versucht, die absurden Symptome des Patienten zu deuten. (Aus *Zelig*.)

Mit Woody Allen (*1935) setzte einer der bedeutendsten Komiker der vergangenen 50 Jahre mit *Zelig* (USA 1983) zwar erneut auf die bewährte Konstellation „männlicher Patient – weiblicher Therapeut“ (Abb. 3), vermied jedoch dank eines grotesk-absurden Plots die typischen Genderklischees; ein Paar wird aus dem Duo dennoch. Allen gelingt mit seiner Komödie eine Gratwanderung, die den bekannten Mustern aus dem Weg geht und als „… filmisches Essay über Identität und Anpassung in der modernen Welt“ [8] Anerkennung fand.

Eine emanzipierte Darstellung findet man in *Agnes of God* (USA 1985, Norman Jewison), wie die oben genannte Produktion *Shadow on the Wall *in der Forensik angesiedelt. Jane Fonda (*1937) verkörpert eine in jeglicher Hinsicht professionelle Psychiaterin, die keine klassische Liebesbeziehung benötigt, um ein Happy End zu finden. Liebe spielt keine Rolle, Komik noch weniger und zum ersten Mal in der Filmgeschichte ist es die Diagnose der Medizinerin, die das Schicksal des Protagonisten zum Guten wendet.

Gegen Ende des 20. Jahrhunderts kam die Psychiatrie endgültig im populären Kino an. So konnten „unter den von 1985 bis 2009 in der Bundesrepublik Deutschland gestarteten Kinofilmen … 522 mit psychiatrierelevanten Motiven identifiziert werden“ [6]. Stigmatisierende Aspekte traten geschlechterübergreifend auf, bei weiblichen Rollen zeigte sich allerdings weiterhin eine sexistische Zuschreibung, die dem alten Stereotyp entspricht: die im Beruf erfolgreiche, aber privat und emotional entwurzelte Psychiaterin. In diesem Sinn verkörpert Nicole Kidman (*1967) in *Batman Forever* (USA 1995, Joel Schumacher) das ironische Comiczerrbild einer Ärztin, die zwar voller Selbstbewusstsein auftritt, aber im eigenen Hedonismus und in aggressiver erotischer Zügellosigkeit schwelgt. Die von Lena Olin (*1955) und Barbra Streisand (*1942) dargestellten Heldinnen in *Mr. Jones *(USA 1993, Mike Figgis) und *Herr der Gezeiten* (USA 1991, Barbra Streisand) besitzen zwar durchaus eine hohe fachliche Expertise, allerdings erneut mit der Folge einer privaten Isolation. In beiden Fällen sorgen die männlichen Protagonisten selbstverständlich für romantische Abhilfe. Eine besondere Bedeutung kommt in *Basic Instinct* (USA, FR 1991, Paul Verhoeven) und in den Filmen der *Lethal-Weapon*-Reihe (USA, 1987–1998) dem Rollenbild der Polizeipsychologin zu: Frauen übernehmen die Funktion eines Korrektivs enthemmter „toxischer Männlichkeit“. Dieser Aspekt kann zudem im Kontext der feministischen Filmtheorie betrachtet werden. Nach Laura Mulveys (*1941) Darlegung gehört die aus der männlichen Perspektive („male gaze“) zum Seh- und Lustobjekt reduzierte Frau nämlich zu den Grundbedingungen des zeitgenössischen Kinos [20]. Demnach könnte auch die Vereinfachung von Rollenbildern in Spielfilmen dieser These gerecht werden.

Trotz hoher fachlicher Kompetenz ist die Darstellung von Professionalität und Berufsethos in der genannten Periode geschlechterübergreifend als negativ einzuschätzen. Die Verletzung des Abstinenz- und Neutralitätsgebots und Grenzüberschreitungen sind ebenso an der Tagesordnung wie offene Feindseligkeit, amouröse Aktivitäten und krankhafte Störungen aufseiten des therapeutischen Personals selbst. Wenn man die narrativen Muster hinsichtlich der eingangs genannten Stereotypentrias bewertet, fällt auf, dass kaum ein Charakter damit ausreichend abgedeckt ist. Vielmehr könnte man mit etwas Fantasie die Kategorie einer weiblichen „Dr. Trapped“ begründen, die sich trotz beruflichem Erfolg und lauterer Absichten heillos in unzulässige private Kontaktaufnahmen mit ihren Patient:innen verstrickt. In jedem Fall belegen diese Motive, dass auch im ausklingenden 20. Jahrhundert die Darstellung von Therapeutinnen fast durchwegs bekannte Stigmaaspekte und Genderklischees mit den erzählerischen Anforderungen des Hollywood-Genrekinos verzahnt.

### Im neuen Jahrtausend

Das dritte Millennium konfrontierte die Filmwelt mit der Entwicklung der Serie. Trotz des Siegeszuges des Fernsehens wurden in das Format der TV-Reihe aber zunächst keine größeren Summen investiert. Bekannte Schauspielerinnen blieben für gewöhnlich beim Film und die Serie fungierte eher als Sprungbrett nach Hollywood. Nach der Jahrtausendwende schwand dieser Unterschied jedoch zusehends. Fortsetzungsgeschichten wie *Lost* (USA, 2004–2010) oder *True Detective* (USA, 2014–heute) mutierten zum starbesetzten Event, das dem Kinoblockbuster den Rang ablief. Entscheidend für diesen Wandel war eine Serie, die heute oft als beste aller Zeiten gehandelt wird [24] und mehrfach im Fokus medizinhistorischer Forschung stand [11, 15]: *Die Sopranos* (USA, 1999–2007).

**Abb. 4:**
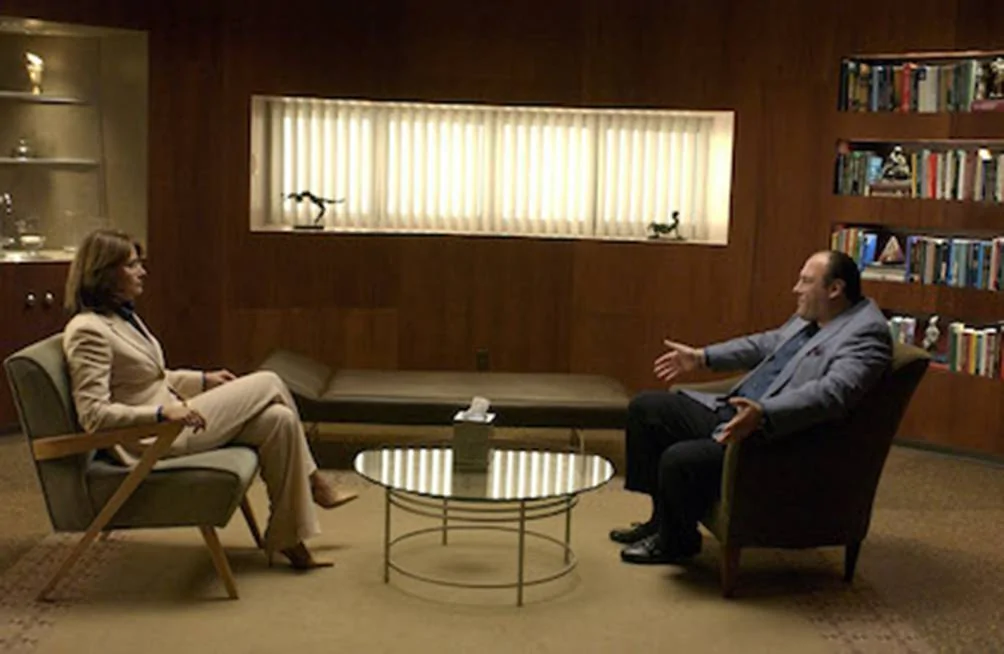
Der Gangsterboss sitzt seiner Psychiaterin gegenüber, die klassische „Psychoanalyse-Couch“ ist aus dem Setting verschwunden. (Aus: *Sopranos*.)

Auch in dieser wegweisenden Produktion findet sich die altbewährte Konstellation aus kompetenter, erfolgreicher Psychiaterin – Dr. Jennifer Melfi – und männlichem, in einem überkommenen Rollenverständnis verhafteten Patienten – Mafiaboss Tony Soprano (Abb. 4). An das bekannte Figurenstereotyp anknüpfend, imponiert die Ärztin als effizient arbeitende, alleinstehende Therapeutin, die ihrem autoritär gesinnten Klienten kompetente Hilfe zukommen lässt. Im Handlungsverlauf zeigen sich durchaus Anzeichen dafür, dass Melfi Sympathien für ihren Patienten hegt. Ob sie sich tatsächlich auch in erotischer Hinsicht von ihm angezogen fühlt, bleibt offen. Statt sich wie ihre „Leinwand-Kolleginnen“ aus früheren Filmen zu riskantem und unprofessionellem Handeln hinreißen zu lassen, erklärt sie ihrem Klienten schlagfertig und einfühlsam, was man unter Übertragung versteht und wie sie zu einer Projektionsfläche für seine Konflikte mit dem weiblichen Geschlecht geworden ist.

Es erscheint bemerkenswert, dass auch die vielleicht einflussreichste Serie der letzten Jahrzehnte eine filmhistorisch höchst klischeehafte Figurenkonstellation aufgreift und nur die Konsequenz ändert: Die Psychiaterin bleibt standhaft. Ob man diesen Umstand als halbherzige Revision strapazierter Geschlechterbilder oder als selbstreferentielle, postmoderne Anspielung auf Stereotypen der Filmgeschichte deutet, bleibt Interpretationssache. In jedem Fall verdeutlicht die Figurenzeichnung, wie nachhaltig etablierte Rollenzuweisungen aus der Filmgeschichte auf die Gegenwart wirken.

## Diskussion und Ausblick

Es überrascht kaum, dass vor Generationen entstandene Filme nicht den heutigen Vorstellungen von Genderkonformität und Geschlechterrollen entsprechen. Verblüffend hingegen erscheint, wie dominant sich ein vorurteilsbehaftetes Psychiaterinnen-Bild in US-amerikanischen Produktionen durch die Geschichte der Cineastik zieht: die kompetent agierende Therapeutin mitsamt ihren weiblich konnotierten Attributen, die in Kombination mit männlichen (Patienten‑)Figuren zu Verfehlungen führen. Zudem wird das Bild einer professionellen Tätigkeit vermittelt, die dem weiblichen Privatleben entgegensteht und ins persönliche Unglück führt. Ebenso scheint die psychoanalytische Couch ein nahezu obligatorisches Motiv zu sein: Filmhandlungen unterscheiden kaum zwischen Psychoanalyse, Psychotherapie und Psychiatrie. Als wesentlicher Faktor für diese Unschärfe ist der enorme Einfluss von Freuds Theorien auf Kultur und Kunstschaffende anzunehmen [26], der vor der Kinematographie keinen Halt machte.

Bedenkt man, dass unglaubwürdige Aspekte wie der früher oft strapazierte Gebrauch von Wahrheitsdrogen rasch aus den Psychiaterinnen-Filmen verschwanden, scheint die Langlebigkeit des filmischen Figurenklischees mehr als ein überkommenes Relikt aus patriarchalisch geprägten Zeiten zu sein. Filmemacher wie Rezipienten störten sich wenig bis gar nicht an solchen Darstellungen. Die Psychiaterin im Film war und ist ohne männlichen Gegenpart nicht denkbar, die Ärztin-Patienten-Beziehung und damit auch die Integrität der Medizinerin läuft stets Gefahr, durch weibliche Emotionen torpediert zu werden. Ausschließlich in ihrer Rolle als Forensikerin und Polizeipsychologin imponiert die fiktive Psychiaterin als unabhängig und durch ihr Geschlecht in keiner Weise kompromittiert. Von *Shadow on the Wall* bis *Basic Instinct *entspricht ihre Funktion der eines Regulativs „toxischer Männlichkeit“ – was im Grunde eine Umkehr der Genderklischees bietet und nun den männlichen Figuren eine „Problemnatur“ unterstellt.

Die Leitfrage, inwiefern Filmpsychiaterinnen der eingangs vorgestellten Figurentrias ihrer maskulinen Pendants entsprechen, kann klar beantwortet werden: Keine der drei Blaupausen lässt sich zwanglos auf die weiblichen Gegenstücke übertragen. Lässt man sich grundsätzlich auf Typisierungen mitsamt den inhärenten Unschärfen ein, kann man dem Trio vielmehr das komplementäre Duo „Dr. Correction“ und „Dr. Trapped“ zur Seite stellen. Allerdings werden die femininen Fiktionen weiterhin nahezu ausnahmslos als Charaktere mit männlichem Gegenpart entworfen, was unterstreicht, wie stark auch heute noch ein genderspezifisches Klischeedenken in den Köpfen der Rezipienten verhaftet ist. Ob und inwiefern diese Denkmuster die Einstellung der Zuschauer gegenüber Psychiaterinnen im echten Leben beeinflussen – insbesondere in Bezug auf Kompetenz und Emotionsregulation –, wirft eine spannende Forschungsfrage für zukünftige Untersuchungen auf. Vergegenwärtigt man sich jedoch, welch immensen Einfluss Filme auf die gesellschaftliche Perzeption von Medizin und Krankheit in der Vergangenheit ausüben konnten [13], drängt sich die Frage auf: Kann Wissen um medial tradierte Klischeebilder im therapeutischen Setting helfen, mögliche Vorbehalte, Ängste und Ressentiments frühzeitig zu erkennen und gezielt zu adressieren? Bestätigt sich diese Hypothese, kann ein Blick in die Psychiatriegeschichte höchst nützlich sein, um Effizienz und Effektivität von Arzt-Patienten-Interaktionen zu optimieren.

## Fazit für die Praxis

- Seit 1935 lässt sich ein stetiger und zuletzt exponentieller Anstieg an Produktionen mit Psychiaterinnen nachweisen, der die reale gesellschaftliche Entwicklung abbildet.
- Schon der Film *Private Worlds* entwirft 1935 erstmals eine stereotype Psychiaterinnen-Figur, die durch weiblich konnotierte Attribute (Einfühlsamkeit, Sensibilität und Emotionalität) Karriere macht, durch die gleichen Eigenschaften jedoch zu unprofessionellem und unethischem Handeln verleitet wird, sobald sie sich in einen Mann verliebt.
- Ausnahmen bilden Psychiaterinnen-Figuren, die in der Forensik oder der Strafverfolgung tätig sind. Hier fungieren sie zumeist als Regulativ „toxischer Männlichkeit“.
- Der Beruf der Psychiaterin wird regelhaft als zeitraubend, nicht familienkompatibel und ins Unglück führend in Szene gesetzt.
- Das Leinwand-Image der Psychiatrie wurde und wird überproportional häufig mit der Psychoanalyse bzw. tiefenpsychologischen Therapie gleichgesetzt.
- Die klassischen Stereotypen der männlichen Psychiater-Figur – Dr. Dippy, Dr. Evil und Dr. Wonderful – passen nicht zu den weiblichen Pendants, für die hier analog zur männlichen Figurentypisierung die Benennungen Dr. Trapped und Dr. Correction vorgeschlagen werden.
- Die Kenntnis des medial bis heute tradierten Psychiaterinnen-Bildes hilft in der Praxis möglicherweise, etwaige Vorurteile gegen eine Behandlung durch Psychiaterinnen nachzuvollziehen und argumentativ abzuschwächen.

## Data Availability

Alle zugrunde liegenden Daten sind im Artikel offengelegt. Zugrunde liegendes Filmmaterial kann aus rechtlichen Gründen nicht öffentlich zugänglich.
